# Macrophage and nerve interaction in endometriosis

**DOI:** 10.1186/s12974-017-0828-3

**Published:** 2017-03-14

**Authors:** Jinjie Wu, Hongyu Xie, Shuzhong Yao, Yanchun Liang

**Affiliations:** 1 Zhongshan School of Medicine, Sun Yat-sen University, Guangzhou, 510089 China; 2 Department of Obstetrics and Gynecology, First Affiliated Hospital of Sun Yat-sen University, No. 58, the 2nd Zhongshan Road, Yuexiu District, Guangzhou, 510080 Guangdong Province China

**Keywords:** Macrophage, Nerve fiber, Neurogenesis, Neuroinflammation, Endometriosis

## Abstract

Dysregulation of the immune system in endometriotic milieus has been considered to play a pivotal role in the pathogenesis of endometriosis. Macrophage recruitment and nerve fiber infiltration are the two major characteristics of this aberrant immune environment. First, the recruitment of macrophages and their polarization phenotype within the endometriotic lesion have been demonstrated to facilitate the development and maintenance of endometriosis. M1 phenotype of macrophages has the capacity to secrete multiple cytokines for inflammatory response, while M2 macrophage possesses an opposite property that can mediate the process of immunosuppression and neuroangiogenesis. Upon secretion of multiple abnormal signal molecules by the endometriotic lesion, macrophages could alter their location and phenotype. These changes facilitate the accommodation of the aberrant microenvironment and the exacerbation of disease progression. Second, the infiltration of nerve fibers and their abnormal distribution are proved to be involved in the generation of endometriosis-associated pain and inflammatory response. An imbalance in sensory and sympathetic innervation and the abnormal secretion of different cytokines could mediate neurogenesis and subsequent peripheral neuroinflammation in endometriosis. Although endometriosis creates an inflammatory milieu promoting macrophage infiltration and an imbalanced innervation, interaction between macrophages and nerve fibers in this process remains unknown. The aim of this review is to highlight the role of macrophage and nerve interaction in endometriosis, where macrophage recruitment and neurogenesis can be the underlying mechanism of neuroinflammation and pathogenesis of endometriosis.

## Background

Endometriosis is defined as the presence of the endometrial-like tissue (lesion) outside the uterus, which is commonly found on the peritoneum, and sometimes as a subsequent invasion into the underlying tissue. It is a common gynecological disease affecting 10% of reproductive age women [1] and is found in up to 20–40% of infertile women [2]. Endometriosis often causes dysmenorrhea, pelvic pain, and infertility, which poses a severe health threat on women and teenage girls’ life [3]. Though scholars have put forward many hypotheses for the pathogenesis of endometriosis [4], the exact mechanism still remains uncertain.

Growing evidence tend to focus on the dysregulation of immune response stimulated by the presence of endometrial debris on the peritoneum of patients with endometriosis [4, 5]. Tissue degeneration of retrograde menstruation plays a central role in triggering inflammatory pain in endometriosis through the activation of innate immune cells and peripheral nerve ending [6]. In their opinion, the activation of the innate immune system is the first important step in the pathophysiology of endometriosis. Macrophages, mast cells, neutrophils, and mature dendritic cells are activated by menstrual debris and subsequently participate in the inflammatory process, while macrophages are the primary contributor of pro-inflammatory chemotactic cytokines and major source of neuroangiogenesis among them [7]. The recruitment and functional changes of macrophages [8], the migration of endothelial cells and subsequent neovascularization [9], and the abnormal generation and distribution of nerve fibers [10] in the lesion are essential events contributing to the dysregulation of endometriosis. Notably, macrophages are important immune cells that can produce both pro-inflammatory and anti-inflammatory cytokines. The recruitment and distribution of macrophages within the endometriotic lesion have been demonstrated to facilitate and maintain endometriosis in patients [11–13]. And nerve fibers in endometriosis also play a crucial role. As the endometriotic lesion develops its own innervation, it is proved to be involved in the generation of pain and inflammation. Imbalance of sensory and sympathetic nerve fibers has also been identified within the endometriotic lesion [14, 15], specifically related to the inflammatory response in the lesion.

Although the infiltration of macrophages and nerve fibers coexist in endometriosis, it remains unknown whether there is a cross-talk between them. Inflammation and neurogenesis are two main factors mediating the pathogenesis of endometriosis. But they are not independent of each other [16]. More importantly, the macrophage can migrate toward the nerve [17], further indicating a specific interaction between the macrophage and nerve in endometriosis. Also, this interaction could be enhanced by estradiol in the peritoneal endometriosis [18]. It is believed that this interaction could probably contribute to the pathogenesis of endometriosis. But the molecular mechanism of this interaction is still elusive. Therefore, it is important to investigate the mechanism of the interaction between macrophages and nerve fibers in endometriosis. Target therapy toward the interaction of macrophages and nerves can not only interfere to the process of inflammation and the activity of peripheral nerves but also remit the progression of endometriosis. The aim of this review is to highlight the potential role of macrophage and nerve interaction in endometriosis in detail. Theoretical elaboration of the underlying mechanism provides a new insight for the pathogenesis of endometriosis.

## Macrophages and their phenotypes in endometriosis

Macrophages are mononuclear phagocytes. Most of them originate from the progenitor in the bone marrow [19]. Once released from the blood vessels, macrophages migrate into the tissue associated with their differentiation into distinct population depending on the anatomical location and the microenvironment of the lesion. Each population of macrophage in specific tissue has a distinct functional profile and gene expression pattern [20]. Different macrophage populations may also exhibit similar functions upon specific stimuli [21], which indicate a remarkable plasticity of macrophages. As an important member of the immune system, macrophages express a wide range of phenotypes, from strong pro-inflammatory responses for elimination of pathogens to anti-inflammatory response for protection and tissue repair. In order to classify the different functions of macrophages, many studies tend to characterize the macrophages as classically or alternatively activated phenotypes based on their receptor composition, secretion profile, and response to the external stimuli [22–24]. Classical activated phenotype, also called M1 macrophages, are activated by interferon gamma (IFN-γ), tumor necrosis factor alpha (TNF-α), or lipopolysaccharide (LPS) [22, 23]. As a consequence, M1 macrophages could produce pro-inflammatory cytokines and chemokines participating in the early stage of injury, pro-inflammatory response, and myoblast proliferation [25]. In contrast, alternatively activated macrophages or M2 macrophages are activated by IL-4, IL-10, IL-13, or transforming growth factor-β (TGF-β) [26, 27]. Once activated, M2 macrophages secrete anti-inflammatory cytokines, growth factors, and other reparative factors [25], which are involved in the anti-inflammatory response, an advanced stage of the repair and healing process. In short, M1 macrophages can kill tumor cells and clear pathogens by activating inflammatory or immune responses, whereas M2 macrophages are immunosuppressive cells that promote tissue repair, tumor angiogenesis, tumor growth, and tumor progression [23].

As mentioned above, macrophages can exhibit high plasticity and different functional profiles. Thus, it is possible for the macrophage to be polarized toward a proper phenotype once stimulated by the signals triggered by the lesion. For example, tumor-associated macrophages (TuAMs) can polarize to M2 phenotype for tumor angiogenesis, growth, and metastasis [28, 29]. With an opposite stimulus, TuAMs can also polarize toward M1 phenotype to exhibit inhibition of tumor growth and progression [30, 31]. This discovery suggests that polarization of the macrophage is probably associated with pathogenesis and progression of the disease.

There have been many studies demonstrating the abnormal distribution of macrophages within the endometriotic lesion of patients with endometriosis [13, 32, 33]. Macrophages in peritoneal fluid and endometriotic lesion can express markers of alternative activated phenotype, from both human specimens and mouse models [34]. Human endometrial macrophages are predominantly M2 macrophages [35]. The abnormal endometriotic milieu can induce the M2 polarization of macrophages leading to the development and invasiveness of endometrial stromal cells [36]. In contrast, one research showed that the ratio of M2 macrophages was significantly lower in the endometriosis group, demonstrating that the macrophage population slants toward M1 in the endometrium of the endometriosis patient [12]. The most likely reason for this discrepancy is the change of macrophage phenotype. In summary, the studies discussed above suggest a novel insight in the polarization of the macrophages in the progression of endometriosis.

## Endometriosis-associated innervation

Innervation associated with the lesion of endometriosis was first demonstrated by Anaf et al. [37], who observed a perineural and interneural invasion in deep infiltrating endometriosis in the recto-vaginal septum. Moreover, there have been several studies demonstrating that the presence and proximity of nerve fibers are related to the associated pain symptom of patients with peritoneal endometriosis [38]. McKinnon et al. revealed that the presence of endometriosis-associated nerve fibers was related to the severity of dysmenorrhea [39]. Further studies have confirmed the presence and high expression of sensory, sympathetic, and parasympathetic nerve fibers in the peritoneal lesion in comparison to the normal peritoneum [40]. In particular, the slow unmyelinated sensory C and the faster myelinated Aδ nerve fibers have also been confirmed in the endometriotic lesion of Sprague-Dawley rats [41] and human samples [40].

Although there is evidence that the presence of nerve fibers has close association with the pain experience in endometriosis patients, many questions still remain unsettled. A great deal of investigators propose that the inflammatory milieu and hyperinnervation are both important factors contributing to the pathophysiology of endometriosis [16]. It is confirmed by Arnold et al. that an increased sensory nerve fiber density (NFD) and decreased sympathetic NFD in a peritoneal endometriotic lesion can be compared to a healthy peritoneum [15]. Additionally, substance P (SP), a member of the tachykinin family that is produced and secreted by sensory nerves, is a strong mediator of neurogenic inflammation [42]. The detection of SP has been confirmed in the peritoneum [40], close to endometriotic lesions [43], as well as deep infiltrating endometriosis (DIE) [44] and peritoneal fluid [45]. Further evidence even suggests that SP has the capacity for the maintenance of the lesion [46]. Instead, peripheral sympathetic neurotransmitters and their co-factors such as norepinephrine, neuropeptide Y, and adenosine have the potential to inhibit important inflammatory functions [47]. These findings imply an interesting speculation that the imbalance of sympathetic and sensory nerve fibers in the endometriotic lesion may result in the disturbance of pro-inflammatory and anti-inflammatory responses.

## Macrophage and nerve interaction in endometriosis

Macrophages and nerve fibers are both important components in endometriosis. It is reasonable to suspect that the macrophage and nerve interaction may mediate the pathophysiology of endometriotic events, including inflammation and hyperinnervation, thereby exacerbating the progression of endometriosis. It is known that M1 and M2 macrophages are correlated to inflammatory and anti-inflammatory responses indicating that macrophage polarization may be important in this interaction.

### Recruitment of macrophages into the lesion of endometriosis

#### Specific molecules in endometriotic milieus recruit macrophages

**Fig. 1:**
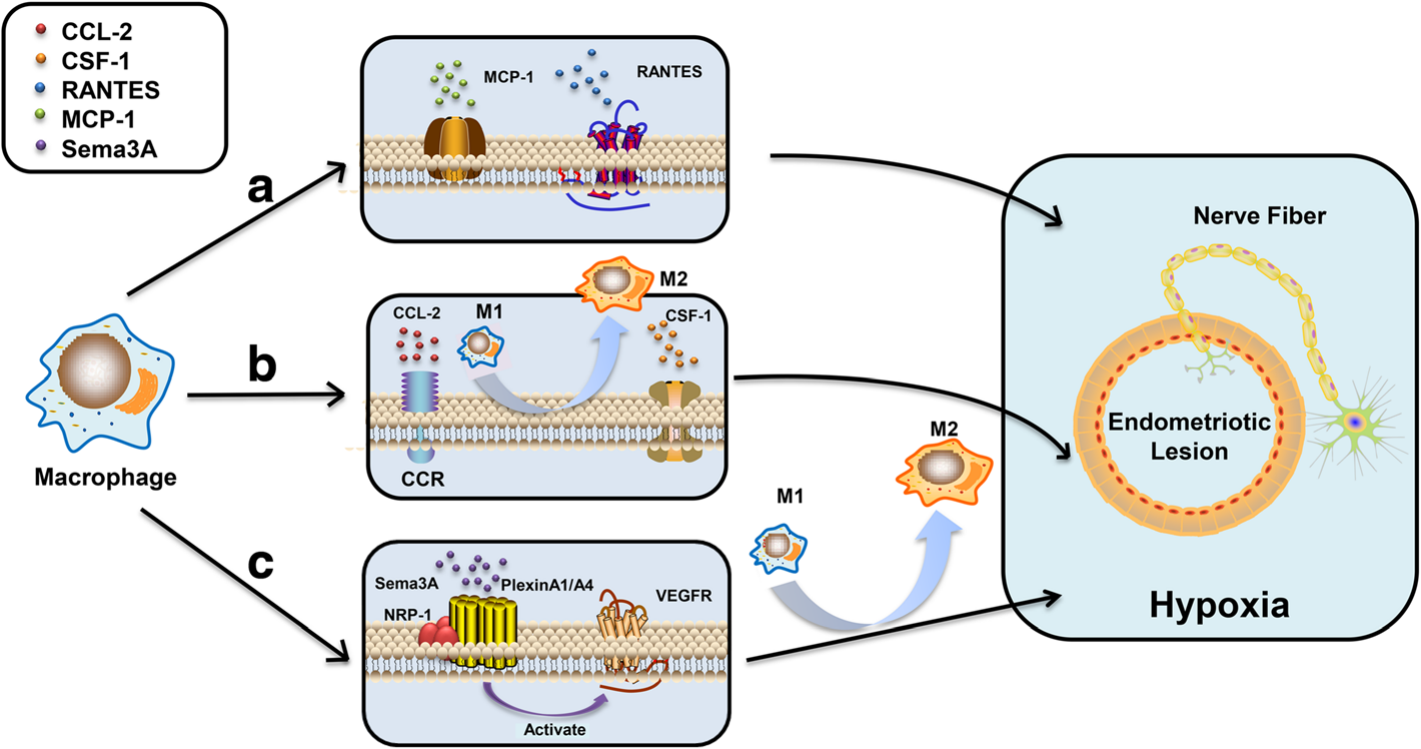
Molecules from the endometriotic lesion recruit macrophages. (*A*) MCP-1 and RANTES in the endometriotic lesion act as attractants to mediate the macrophage recruitment. (*B*) CCL-2 and CSF-1 secreted from nerve fibers recruit the macrophages into the endometriotic milieu. The CCL-2-mediated recruitment also polarizes the macrophages toward M2 phenotypes. (*C*) Sema3A/NRP-1 signaling guides the macrophages to the hypoxic microenvironment and regulates the polarization of macrophages toward M2 phenotype. CCL-2: chemokine (C–C motif) ligand 2 or monocyte chemoattractant protein 1 (MCP-1); RANTES: C–C chemokine, regulated on activation, normal T cell expressed and secreted; CCR2: C–C chemokine receptor type 2; CSF-1: colony-stimulating factor 1; Sema3A: semaphorin 3A; NRP-1: neuropilin-1; PlexinA1/A4: plexinA1/A4 receptor; VEGFR: vascular endothelial growth factor receptor; M1: classical activated macrophage; M2: alternatively activated macrophage

Since the temporal distribution of macrophages and hyperinnervation contribute to the development of endometriosis, an understanding of the regulatory mechanism of the recruitment of the macrophage into the endometriotic lesion can provide a comprehensive mechanism of the disease progression. Numerous molecules with aberrant expression in the endometriotic lesion have been identified to be responsible for the recruitment of macrophages. An increased secretion of monocyte chemotactic protein-1 (MCP-1, also known as chemokine ligand 2, CCL-2) from peritoneal macrophages of women with endometriosis may contribute to paracrine and autocrine activation, leading to macrophage accumulation in the peritoneal cavity of patients with endometriosis [48]. Further studies have confirmed that endometriosis-induced expression of MCP-1 enhanced macrophage recruitment in the human body or in rat models [8, 49]. In addition to MCP-1, Wang et al. directly demonstrated that the increased RANTES (regulated on activation, normal T cell expressed and secreted) from the endometriotic focus-associated cells could recruit macrophages into the ectopic milieu [50]. In addition, a high level of RANTES can also induce macrophage tolerance. Vitamin D receptor (VDR) has been confirmed to be expressed both in eutopic and ectopic endometrium [51]. Agonist of VDR inhibits lesion development accompanied with the inhibition of macrophage recruitment, which has implied an effect of VDR on macrophage migration in endometriotic milieus [52]. Other chemokines such as IL-8 [53] in endometriotic milieus also have capacity for macrophage recruitment [54] (Fig. 1(A)).

#### Attractants from nerve fibers recruit macrophages

Furthermore, the presence of the nerve fibers also provides a new pattern for the recruitment of macrophages. More inflammatory cells are observed near nerve fibers in women with endometriosis, suggesting a direct attraction of macrophages to nerve fibers [39]. Greaves et al. [18] have proved that colony-stimulating factor 1 (CSF-1) and CCL-2 secreted from nerves are attractant factors for macrophages, verifying the role of CSF-1 and CCL-2 on the enhancement of the macrophage migration. More evidence even demonstrated that CCL-2/CCR-mediated migration could also polarize macrophages from M1 toward M2 phenotype [55]. Besides the factors discussed above, many other factors have been demonstrated to be involved in mediating the macrophage recruitment toward the nerve within the lesion, including leukemia inhibitory factor (LIF) [56], IL-1α, IL-1β [57], and pancreatitis-associated protein III (PAP-III) [58] (Fig. 1(B)).

#### Sema3A/NRP-1 signaling mediates macrophage recruitment

Semaphorins are a family of surface or locally secreted proteins that are evolutionarily highly conserved. Sema3A, a secreted class 3 semaphorin, is an axon-repulsive guidance factor for neuron. It is characterized by its function on axonal elongation restriction and growth cone collapse through binding to a multimeric holoreceptor complex, neuropilin-1 (NRP-1) [59], and PlexinA1 [60, 61]. Recently, Sema3A has been detected in peritoneal and deep infiltrating endometriosis, and it is proved to play a potential role in mediating the aberrant sympathetic innervation in the lesion [62]. There are many studies demonstrating that Sema3A/NRP-1 signaling not only mediates macrophage migration but also regulates the polarization of macrophages. Casazza et al. [63] found that NRP-1 expression was downregulated on the surface of tissue-associated macrophages (TiAMs) in the hypoxic environment, whereas NRP-1 level was higher in a normoxic environment. This phenomenon indicated that the location of TiAM was tightly controlled by Sema3A/NRP-1 signaling. This study also demonstrated that Sema3A and its receptors mediate the migration through activation of vascular endothelial growth factor receptor (VEGFR). Similar results even suggested that Sema3A/NRP-1 signaling was involved in the macrophage reprogramming [64]. Expression of Sema3A and its receptors have been identified on monocyte-derived macrophages [65]. M1 and M2 macrophages can be distinguished by different patterns of NRP-1 and PlexinA1. These findings suggest a potential mechanism of macrophage recruitment that Sema3A/NRP-1 signaling guides the macrophages toward the hypoxic environment of endometriosis, accompanied with the changes of polarization toward M2 phenotype. M2 macrophages with decreased NRP-1 in a hypoxic lesion are more likely to escape from the negative effect of repellent factors (such as Sema3A), resulting in M2 macrophage accumulation which leads to angiogenesis, proliferation, and progression (Fig. 1(C)). Thus, it is reasonable to speculate that Sema3A may be a potential medium to regulate the progress of macrophage migration and the following interaction with nerve fibers in the lesion of endometriosis.

### Macrophages and neurogenesis in endometriosis

As a matter of fact, neural hypertrophy has been detected in patients with severe pain experience. There are many studies supporting that pain sensation of endometriosis patients is correlated with neural hypertrophy and increased neural density in the endometriotic lesion [66, 67]. Previously it has been confirmed that the density of nerve fibers in the peritoneal endometriotic lesion is higher than that in the peritoneum from women without endometriosis [40]. The higher density of nerve fiber was even observed in the deep-invasive lesion involving the bowel [68]. Considering the imbalance of sympathetic and sensory innervation in endometriosis, the disturbance of neurogenesis of sympathetic and sensory nerves may occur and contribute to the progression of endometriosis, especially to the generation of neuropathic pain [2].

#### Neurotrophins from macrophages mediate neurogenesis in endometriosis

Neurotrophins are a family of proteins that is important for the development, maintenance, survival, and differentiation of neurons in both the central nervous system and peripheral nervous system [69]. They belong to a class of growth factors and secreted proteins, including nerve growth factor (NGF), brain-derived neurotrophic factor (BDNF), neurotrophin-3 (NT-3), and neurotrophin-4 (NT-4) [70]. The expression of the neurotrophin family is not only investigated in endometriosis [71–74] but also widely documented in macrophages by multiple studies [75–77]. NGF mRNA was overexpressed in ovarian endometriosis and deep infiltrating endometriosis, significantly higher than that of eutopic endometrium and control [78]. Barcena et al. [73] also provided evidence for the participation of neurotrophin in the endometriosis-associated innervation. They confirmed that the expression of NGF and NT-3 were increased significantly in the peritoneal fluid (PF) of patients with endometriosis, and BDNF was also detected in PF of women with peritoneal endometriotic lesion. Moreover, this study further revealed an increased sensory neurite outgrowth and a decreased sympathetic neurite outgrowth once incubated with PF and NGF. These findings distinctly verify the role of neurotrophins in nerve fiber outgrowth and imbalance of sensory and sympathetic nerve fibers in endometriosis. Recently, some investigators have found out the cellular source of neurotrophins, and they demonstrated that the upregulation of NT-3 and BDNF were induced by macrophage in an estradiol-dependent manner [18], contributing to the neurogenesis in the endometriotic lesion. Many other reports also confirm that macrophages are the important source of NGF [79], which is necessary for nerve sprouting and reorganization of sensory and sympathetic nerve fibers. These evidence provide the fact that macrophages may participate in the process of neurogenesis by the secretion of neurotrophins in the endometriotic lesion.

#### Semaphorins from macrophages mediate neurogenesis in endometriosis

In addition to the neurotrophins, there are many other proteins which participate in the neurogenesis of endometriosis. They are proteins for axonal guidance including semaphorins and their plexin and neuropilin receptors [62], slit ligands and their roundabout (ROBO) receptors [80], and ephrins and their Eph receptors [81], etc. The axon-repulsive guidance factor, Sema3A, has been detected in the endometriotic lesion [62], and the regulation of Sema3A by M2 macrophage is already confirmed [82]. However, the co-existence of a nerve-repellent factor (Sema3A) and a nerve-promoting factor (NGF) in the lesion seems to be inconsistent with the nerve-sprouting phenomenon. It has been proposed that decreased Sema3A and increased NGF expression might trigger the outgrowth of C-fibers [83]. The increased NGF concentration can abolish Sema3A-induced inhibitory effect on axon outgrowth [84]. More evidence about this conflict are provided that Sema3A induces a repulsive effect on the sensory nerve and may even induce cell death in the NGF-dependent dorsal root ganglion (DRG) neuron [85], while NGF could induce sensory nerve outgrowth and suppress the growth of sympathetic nerve [73]. These evidence solve the discrepancy between Sema3A and NGF and indicate that NGF and semaphorins in the endometriotic lesion have an inter-restricted relationship. Disturbance of this relationship would lead to the imbalance of sensory and sympathetic innervation.

#### Macrophages regulate neurogenesis in endometriosis

**Fig. 2:**
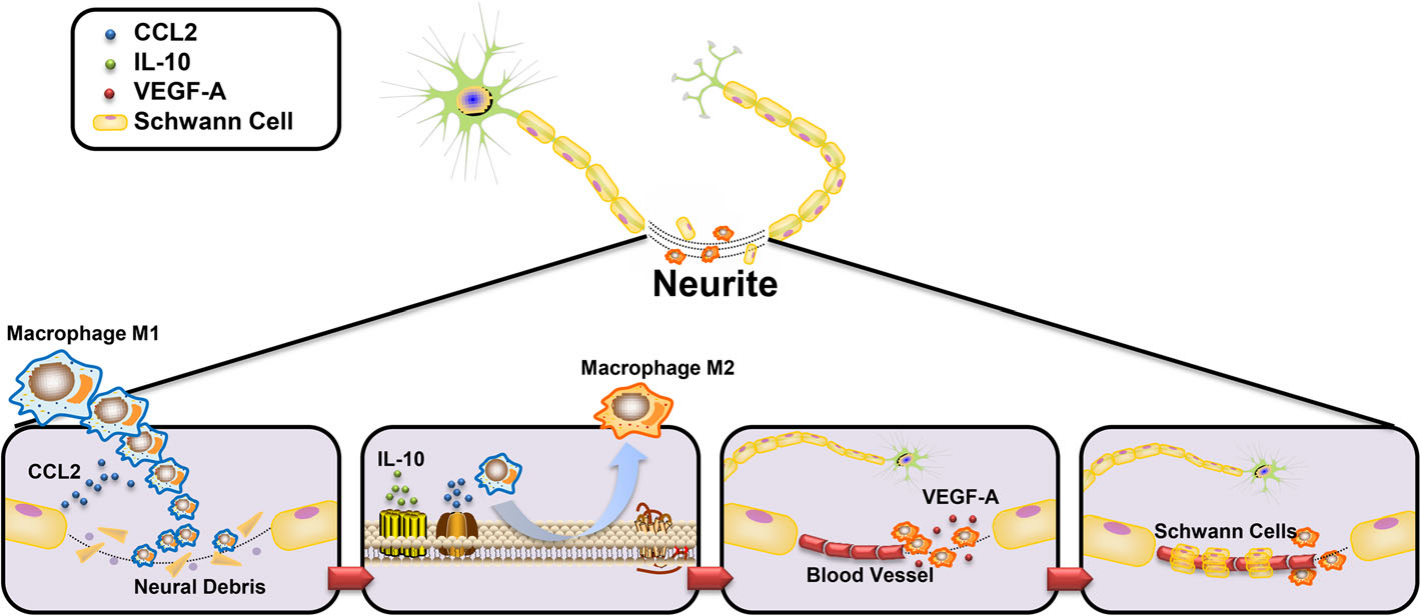
Macrophages regulate neurogenesis after peripheral nerve injury. After peripheral nerve injury happens in endometriosis, macrophages are firstly attracted to the lesion by CCL-2 secreted from Schwann cells. After the clearance of neural debris, M1 macrophages polarize toward M2 phenotype mediated by the CCL-2 and IL-10 in the milieus. M2 macrophages release VEGF-A, promoting the formation of blood vessels. After that, the Schwann cells migrate through the lesion along the blood vessels and then guide the axonal regeneration. Therefore, the regeneration of neurites is initiated and mediated by activities of macrophages. CCL-2: chemokine (C–C motif) ligand 2; IL-10: interleukin 10; VEGF-A: vascular endothelial growth factor A

Most of the proteins discussed above could be secreted by macrophages, implying the role of macrophages in neurogenesis in endometriosis. The necessary role of the macrophage is enhanced in studies that focus on sympathetic sprouting. The inhibition of the Notch signaling pathway could attenuate NGF-induced sympathetic nerve sprouting by polarizing the macrophages toward M2 phenotype [86]. Accordingly, we can speculate that there exists a similar association between macrophage polarization and neurogenesis in endometriosis. Upon peripheral nerve injury, macrophages are the major immune cells removing myelin and axonal debris [87]. These recruited macrophages in the hypoxic microenvironment of endometriosis are then polarized toward M2 phenotype [88]. They subsequently activate the formation of blood vessels by releasing VEGF-A into the lesion, which helps the migration of Schwann cells, facilitating peripheral axon regeneration [89]. Although the mechanism underlying the neurogenesis in endometriosis has not been confirmed, peripheral nerve injury-induced neurogenesis is proposed to be of great importance. Scholars consider that endometriosis may be either the consequence of uterine denervation and reinnervation [90] or the consequence of varying injuries to pelvic autonomic nerves [91]. Thus, the polarization of macrophages and the interaction between the nerve and macrophage in PNS regeneration provide an important insight into how macrophages participate in neurogenesis in the endometriotic milieu (Fig. 2).

### Macrophage and nerve fiber interaction contributes to neuroinflammation and pain generation in endometriosis

It is well known that the progression of the endometriosis could promote the activity of the immune cells and thereby stimulate the inflammatory response [92]. The increased secretion of inflammatory cytokines [7], chemokines [93], and growth factors [94, 95] in endometriosis has been widely demonstrated. Although the presence of endometriotic pain is mostly associated with the disease progression [96], the generation of the pain in endometriosis remains unknown. Recently, more and more studies focus on the relation between inflammation and pain experience [16, 66]. Focal inflammation stimulates the peripheral nerve ending, and the activated nerve fibers also secrete pro-inflammatory neuromodulator, which is called peripheral neuroinflammation. More evidence support that peripheral neuroinflammation may contribute to hypersensitivity and hyperalgesia of sensory neurons [97]. Peripheral neuroinflammation is an inflammation process characterized by the recruitment of macrophages [98, 99] and infiltration of nerve fibers [100].The trigger of inflammation by macrophages and pro-inflammatory peptides released by nerve fibers may form a vicious cycle between macrophages and nerve fibers. It may be the mechanism of maintenance and aggravation of inflammation and generation of pain.

It has been previously discussed that macrophages and nerve fibers are both detected in the endometriotic milieus. Multiple cytokines from macrophages can stimulate peripheral nerve sensitization. TNF-α and MCP-1, mainly secreted by macrophages, are highly expressed in the endometriotic lesion [101, 102], which are also mainly secreted by macrophages. They can induce the sensory nerve to produce a sustained induction of action potential via transient receptor potential vanilloid 1 (TRPV1) [103, 104]. Voltage-gated sodium channels are encoded by SCNA genes, which are highly expressed in women with endometriosis [105]. There have been many studies confirming that TNF-α and MCP-1 also have the ability to modulate the voltage-gated sodium channels by enhancing the currents [106–108], which may lead to hypersensitivity and hyperalgesia.

**Fig. 3:**
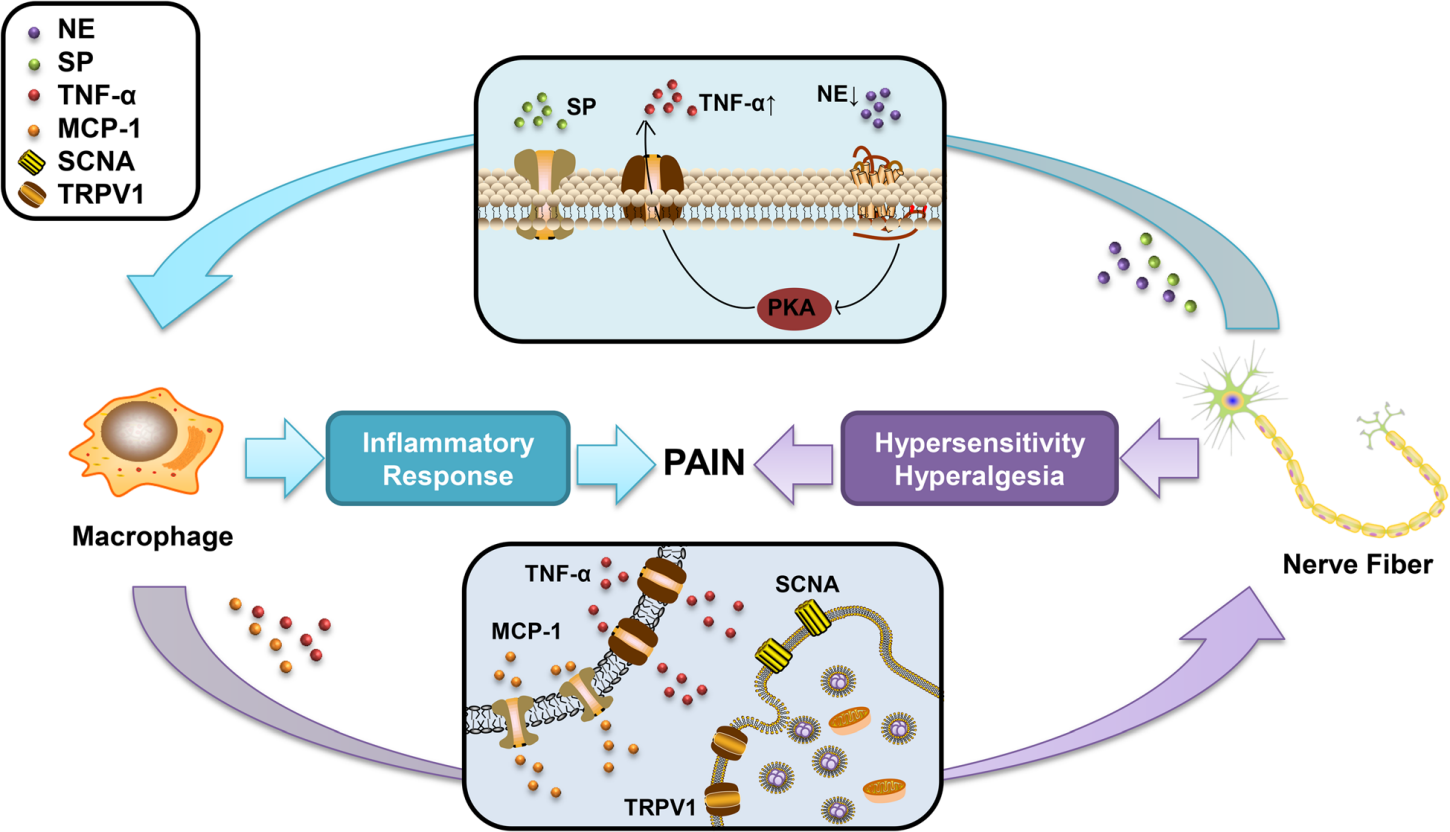
Macrophage and nerve fiber interaction contributes to inflammatory response and hypersensitivity in endometriosis. NE from decreased sympathetic nerve fibers binds the β2 receptor on macrophages and then activates the PKA signaling leading to the increased TNF-α from macrophages and therefore regulating the inflammatory response. SP released from sensory nerve fibers stimulates the macrophage to release pro-inflammatory cytokines. TNF-α and MCP-1 secreted from macrophages bind to SCNA and TRPV1 on the surface of the neuron. This process enhances the currents in the nerve fibers and leads to hypersensitivity and hyperalgesia. Both inflammation and hypersensitivity in the endometriotic lesion contribute to the generation of pain sensation. NE: norepinephrine; TNF-α: tumor necrosis factor alpha; SP: substance P; MCP-1: monocyte chemoattractant protein 1; SCNA: sodium channel protein; TRPV1: transient receptor potential vanilloid 1

On the other hand, transmitters from sympathetic nerve fibers, such as norepinephrine (NE), adenosine (AD), and opioids, possess anti-inflammatory properties [109]. Tang et al. have confirmed the mechanism of NE-induced anti-inflammation [110]. NE from sympathetic nerve fibers binds the β2 receptor on macrophages and then activates the PKA signaling leading to the decreased TNF-α from macrophages. Decreased sympathetic nerve fibers may stimulate TNF-α secretion from macrophages, mediating the inflammation in endometriotic lesion. The neuropeptide SP appears in the sensory nerve fibers of peritoneal and deep infiltrating endometriostic lesion. SP can stimulate macrophages and induces the release of pro-inflammatory cytokines, such as TNF-α, IL-6, and IL-8 [111]. These molecules have the ability to enhance the inflammation in the microenvironment. Therefore, decreased sympathetic nerves and increased sensory nerves both promote pro-inflammatory effects, suggesting that there exists a neurogenic inflammatory process in the endometriotic lesion (Fig. 3).

In general, nerve transmitters and mediators could alter the status of macrophages for accommodation of the inflammatory endometriotic milieus, while macrophages could release the cytokines responding to the inflammation and subsequently change the excitability of the sensory nerve. The existence of the vicious cycle between macrophages and nerve fibers in endometriosis plays an important role to maintain neuroinflammation and aggravate the inflammation-induced pain. Furthermore, more studies are needed to investigate the role of macrophage and nerve interaction in mediating these processes.

## Conclusions

**Fig. 4:**
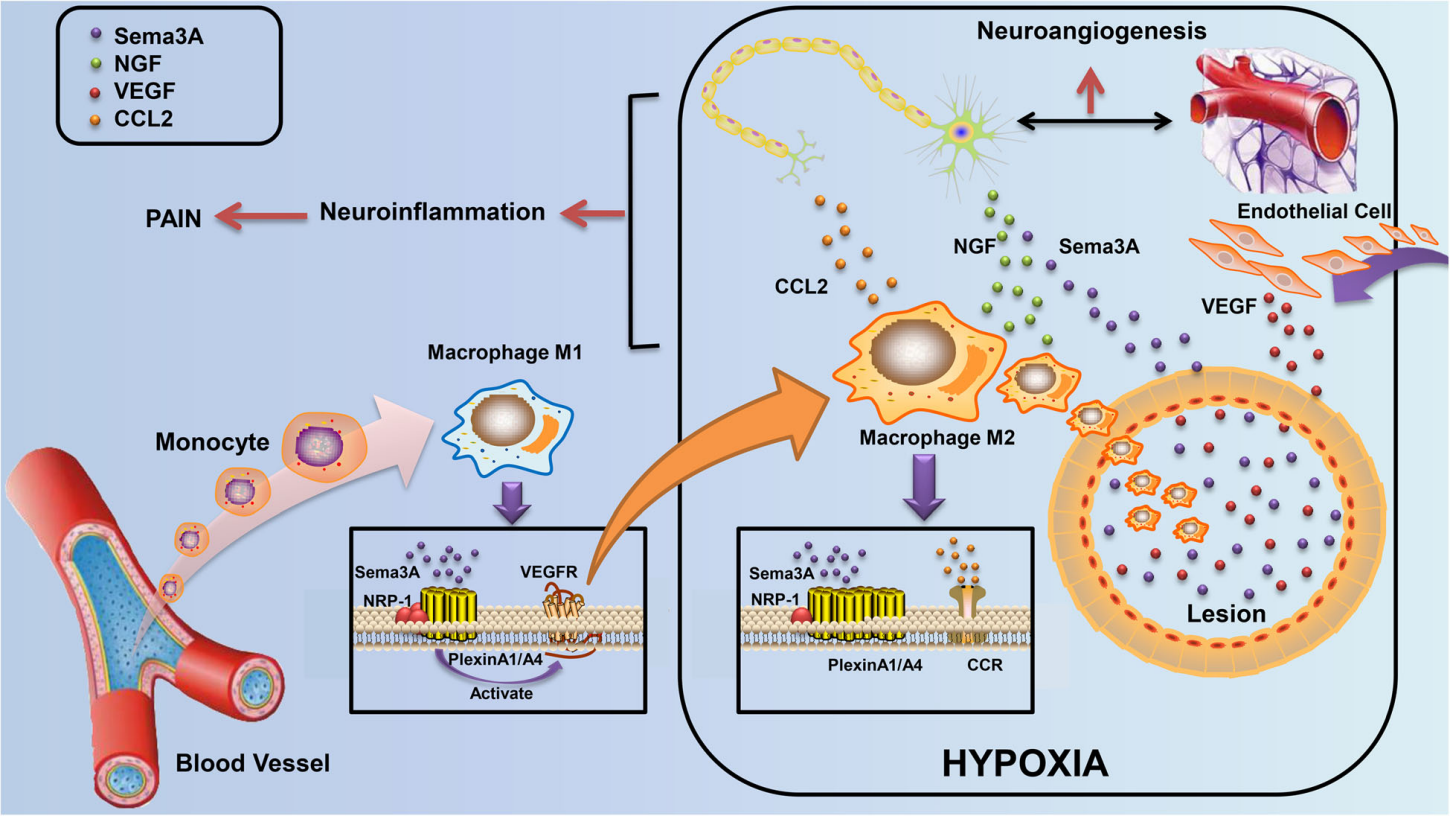
Macrophage polarization and aberrant nerve distribution participate in peripheral neuroinflammation and neuroangiogenesis in endometriosis. Monocytes emigrated from the blood vessels can differentiate into macrophages under different stimuli. After that, macrophages migrate into the endometriotic lesion, mediated by multiple specific molecules (such as CSF-1, CCL-2, and MCP-1) in endometriosis. Sema3A/NRP-1 regulates the polarization of macrophage phenotype and causes macrophage entrapment into the lesion simultaneously. M2 macrophages in the lesion can secrete Sema3A, NGF, and VEGF, participating in the neuroangiogenesis in endometriotic milieus. Nerve fibers in the lesion are also capable to regulate the activity of macrophages. Macrophage and nerve interaction may exacerbate neuroinflammation in endometriosis, eventually resulting in the occurrence of pain sensation

Endometriosis is a very complex gynecological condition characterized by its frequent association with debilitating pelvic pain, dysmenorrhea, dyspareunia, and infertility. These symptoms impact their relationships, their work, and their health-related quality of life for months or even years. Although the pathogenesis of endometriosis is not clear entirely, inflammation and neurogenesis in the endometriotic milieus are now considered to contribute to the progression of the disease, especially the generation of pain experience [16]. As retrograde menstruation promotes the inflammatory microenvironment, macrophage infiltration and hyperinnervation are both important events mediating the development of endometriosis. Initially, macrophages migrate into the lesion in response to the molecular changes in endometriotic milieus, including multiple chemoattractants from nerve fibers. This migration is associated with the phenotype changes which could facilitate the entrapment of macrophages in the lesion. Subsequently, macrophages in the lesion can secrete proteins that have neuroprotective properties, promoting the outgrowth of nerve fibers. Since both macrophages and nerve fibers can induce inflammatory response, the interaction between macrophages and nerve fibers in endometriosis is considered to be the foundation of neuroinflammation and eventually results in the hypersensitivity and hyperalgesia of sensory nerves (Fig. 4).

However, current medical guidelines poorly reflect the complexity of inflammation in the lesion, which only interferes with inflammation and pain by suppressing the lesion and modulating the menstrual cycle indirectly [112]. Due to the molecular nature of endometriosis, the limited efficacy of currently available hormonal drugs, and their potential adverse effects, there is an urgent medical need for innovative and more effective treatment. Based on the central role of the interaction between macrophages and nerve fibers, a promising target involved in the interference of both macrophages and stimulation of peripheral nerves may be developed. Prostaglandin E2 can impair phagocytic ability of peritoneal macrophages via suppression of annexin A2 [113]. Glucosaminyl muramyldipeptide, an immunomodulatory drug, can also prevent hyperactivation of macrophages [114]. Antiangiogenic treatment statistically diminishes macrophages, mast cells, and nerve fibers in mice and therefore alleviates severe pelvic pain associated with human peritoneal endometriosis [115]. Although all of the drugs mentioned above are intended to relieve the inflammation in endometriosis, compounds that act on the pathway in the interaction of macrophages and nerve fibers will have the most significant therapeutic potential, such as Sema3A, MCP-1, or TNF-α. Furthermore, there is still an urgent need that we know more about the interaction between macrophages and nerve fibers. Actually, this theoretical elaboration provides a new insight for the therapeutic strategy in endometriosis. Targeting macrophages and nerve fibers may potentially regulate neuroinflammation and even disrupt the pain occurrence in women with endometriosis.

## Abbreviations

AD: Adenosine
BDNF: Brain-derived neurotrophic factor
CCL-2: Chemokine ligand 2
CSF-1: Colony-stimulating factor 1
DIE: Deep infiltrating endometriosis
DRG: Dorsal root ganglion
IFN-γ: Interferon gamma
LIF: Leukemia inhibitory factor
LPS: Lipopolysaccharide
MCP-1: Monocyte chemotactic protein-1
NE: Norepinephrine
NFD: Nerve fiber density
NGF: Nerve growth factor
NRP-1: Neuropilin-1
NT-3: Neurotrophin-3
NT-4: Neurotrophin-4
PAP-III: Pancreatitis-associated protein III
PF: Peritoneal fluid
RANTES: Regulated on activation, normal T cell expressed and secreted
ROBO: Roundabout
SCNA: Voltage-gated sodium channel genes
Sema3A: Semaphorin 3A
SP: Substance P
TiAMs: Tissue-associated macrophages
TuAMs: Tumor-associated macrophages
TGF-β: Transforming growth factor-β
TNF-α: Tumor necrosis factor alpha
TRPV1: Transient receptor potential vanilloid 1
VDR: Vitamin D receptor
VEGF: Vascular endothelial growth factor
